# Changes in vascular permeability in the spinal cord contribute to chemotherapy-induced neuropathic pain

**DOI:** 10.1016/j.bbi.2019.10.018

**Published:** 2020-01

**Authors:** Karli Montague-Cardoso, Thomas Pitcher, Kim Chisolm, Giorgia Salera, Erik Lindstrom, Ellen Hewitt, Egle Solito, Marzia Malcangio

**Affiliations:** a Wolfson Centre for Age-related Diseases, Guy’s Hospital Campus, King’s College London, London SE1 1UL, United Kingdom; b William Harvey Research Institute, Bart’s and The London School of Medicine Queen Mary, Charterhouse Square, London EC1M 6BQ, United Kingdom; c Medivir AB, Huddinge, Sweden

**Keywords:** Chemotherapy-induced neuropathic pain, Vincristine, Monocytes, Permeability, Blood-spinal cord barrier, Endothelium, Infiltration, Tight Junctions, Cathepsin S

## Abstract

Chemotherapy-induced neuropathic pain is a dose-limiting side effect of many cancer therapies due to their propensity to accumulate in peripheral nerves, which is facilitated by the permeability of the blood-nerve barrier. Preclinically, the chemotherapy agent vincristine (VCR) activates endothelial cells in the murine peripheral nervous system and in doing so allows the infiltration of monocytes into nerve tissue where they orchestrate the development of VCR-induced nociceptive hypersensitivity. In this study we demonstrate that VCR also activates endothelial cells in the murine central nervous system, increases paracellular permeability and decreases trans endothelial resistance. In *in vivo* imaging studies in mice, VCR administration results in trafficking of inflammatory monocytes through the endothelium. Indeed, VCR treatment affects the integrity of the blood-spinal cord-barrier as indicated by Evans Blue extravasation, disrupts tight junction coupling and is accompanied by the presence of monocytes in the spinal cord. Such inflammatory monocytes (Iba-1^+^ CCR_2_^+^ Ly6C^+^ TMEM119^-^ cells) that infiltrate the spinal cord also express the pro-nociceptive cysteine protease Cathepsin S. Systemic treatment with a CNS-penetrant, but not a peripherally-restricted, inhibitor of Cathepsin S prevents the development of VCR-induced hypersensitivity, suggesting that infiltrating monocytes play a functional role in sensitising spinal cord nociceptive neurons. Our findings guide us towards a better understanding of central mechanisms of pain associated with VCR treatment and thus pave the way for the development of innovative antinociceptive strategies.

## Highlights

• Vincristine (VCR) induced nociception can be dose-limiting.
• VCR increases permeability of the blood-spinal cord barrier.
• Increased permeability results in infiltration of monocytes into the spinal cord.
• Infiltrating monocytes express the pronociceptive enzyme Cathepsin S (CatS).
• A centrally-penetrant CatS antagonist reduces VCR-induced nociception.

## 1 Introduction

Neuropathic pain results from central nervous system (CNS) and peripheral nervous system (PNS) lesions that are accompanied by significant immune responses, such as those elicited by monocytes/macrophages and microglia (CNS-resident macrophages). These immune cells produce inflammatory mediators that contribute to neuropathic pain by activating nociceptive neurons (Costigan et al., 2009). The mechanisms that govern the involvement of immune cells however, differs between the CNS and PNS.

Injury in the CNS such as that caused by traumatic spinal cord lesions can result in disruption of the integrity of the blood–brain barrier and the consequential infiltration of inflammatory monocytes, which express the chemokine receptor CCR_2_. Such infiltration, along with microglial responses, exacerbates neuronal damage and pain. For instance, in CNS conditions such as spinal cord injury, CCR_2_^+^ monocytes infiltrate the dorsal column and facilitate neuronal damage (Evans et al., 2014). Here they are recruited by the chemokine CCL_2_, which is expressed by perivascular macrophages and microglia, and contribute to inflammation (Varvel et al., 2016).

In the instance of PNS injury however, the role of monocytes and macrophages has been shown to remain peripheral. Resident macrophages in the dorsal root ganglia (DRG) produce soluble factors, which induce the recruitment of neutrophils and monocytes (Santa-Cecília et al., 2019). In addition, as a consequence of endothelial cell activation, monocytes are able to infiltrate into peripheral nerves following PNS injury, where they orchestrate the development of mechanical hypersensitivity (Old et al., 2014). This however, is not mirrored centrally where it is the microglial that respond to injury-induced increases in nociceptive neuron activity. For instance, microglia release the cysteine protease Cathepsin S (CatS) that solubilises the chemokine domain of neuronal fractalkine (FKN), which in turn activates the CX_3_CR_1_ chemokine receptor in microglia (Clark et al., 2007, Clark et al., 2009). FKN activation of CX_3_CR_1_ results in the release of pro-nociceptive cytokines, such as IL-1β (Clark et al., 2015, Innes et al., 2019), that induce neuronal sensitisation and facilitates pain signalling as well as other pathological conditions.

Peripheral nerve injury manifests during and after treatment with a variety of chemotherapeutic agents, which have a broad range of actions with both shared and drug-specific underlying mechanisms. The blood-nerve barrier is relatively permeable as a result of low activity of the P-glycoprotein transporter (Balayssac et al., 2005). Chemotherapuetic agents consequently accumulate in peripheral nerves and cause neurotoxicity associated with monocyte infiltration/resident macrophage activation in peripheral nerves and the DRG, as well as significant nociceptive hypersensitivity (Old et al., 2014, Zhang et al., 2016, Shen et al., 2017, Montague et al., 2018). For example, in the case of hyperalgesia induced by oxaliplatin, a higher concentration of the drug is found in the DRG compared to the spinal cord and is critical for activation of resident macrophages (Shen et al., 2017). Another chemotherapeutic agent, which results in painful peripheral neuropathy is the vinca alkaloid vincristine (VCR). VCR treatment is associated with significant nociception, which is orchestrated by monocyte infiltration into peripheral nerves. Specifically, VCR treatment induces the expression of the adhesion molecules ICAM-1 and VCAM-1 in endothelial cells and consequently promotes the infiltration of patrolling monocytes (CX_3_CR_1_^high^ monocytes) into the sciatic nerve, later followed by inflammatory monocytes (CCR_2_^high^/CX_3_CR_1_^mid^) as treatment progresses. Both patrolling and inflammatory monocytes facilitate noxious signalling through the release of cytokines and reactive oxygen species (ROS), which sensitise sensory neurons (Old et al., 2014, Montague et al., 2018). Indeed, pharmacological impairment of monocyte trafficking in peripheral nerves both prevents and reverses VCR-induced mechanical hypersensitivity (Montague and Malcangio, 2017). Importantly, whilst monocytes/macrophages play a mechanistic role in pain associated with VCR treatment, changes in microglial number and morphology centrally and are not apparent when clinically-relevant doses of VCR are administered, as is the case with other chemotherapeutic agents (Zheng et al., 2011, Old et al., 2014).

The clinical preparation of VCR – vincristine sulphate, is a large molecule (933 g/mol) which, despite passing through the peripheral blood-nerve barrier, does not accumulate centrally as it does not cross through the blood–brain barrier (BBB) or blood-spinal cord barrier (BSCB) where P-glycoprotein transporter efficiently limits drug distribution (Balayssac et al., 2005, Wang et al., 2010). The endothelial cells that form both the BBB and BSCB are tight junction-coupled. Proteins that constitute tight junctions include claudins, which form part of the extracellular component, and zona occludens proteins, which are one of the building blocks of the cytoplasmic component of the junction (Findley and Koval, 2009). The presence of tight junctions effectively restricts paracellular diffusion (Abbott et al., 2010).

In this study we explored whether VCR activates endothelial cells in the CNS and alters the integrity of the BSCB. We expand our knowledge regarding the role of monocyte signalling in VCR-induced nociception and begin to uncover a role for monocytes that infiltrate into the spinal cord as an indirect result of VCR treatment. Specifically, we uncover what we believe to be a previously unidentified alteration in vascular permeability of the BSCB that is mediated by VCR. We also investigate the effect of treatment with inhibitors of CatS on VCR-induced nociception. We use both peripherally-restricted and centrally-penetrant CatS inhibitors in order to assess the importance of central *versus* peripheral monocyte-derived CatS-regulated mechanisms. Our findings guide us towards a better understanding of central mechanisms of pain associated with VCR treatment and thus pave the way for the development of innovative therapeutic strategies.

## 2 Materials & methods

### 2.1 Animals

Experiments were performed in accordance with the United Kingdom Animals (Scientific Procedures) Act 1986 and local animal care and use guidelines. All mice were housed under a 12 h light/dark cycle, with food and water available *ad libitum*. For behavioural experiments both male and female cx3cr1-gfp (heterozygous and thus normal cx3cr1 function) were used (25–30 g, 16 weeks of age). For Evans Blue experiments C57Bl/6 male and female mice (25–30 g, 12–17 weeks of age) were used. For intravital experiments and immunohistochemistry CCR_2_-RFP (red fluorescent protein) mice were used for which an original breeding stock was purchased from Jackson Laboratory having been generated from a C57BL/6 background. CCR_2_ disruption/RFP expression was confirmed by PCR using published primers (Montague et al., 2018).

### 2.2 Behavioural testing

Static mechanical withdrawal thresholds were assessed as previously described (Pitcher et al., 2016). Briefly, unrestrained mice were acclimatised in custom-made chambers for 1 h prior to testing. Calibrated von Frey filaments (0.008–1.04 g) were gently applied to the hind paw (plantar surface) until they started to bend and were held in place either for 3 s or until the paw was withdrawn. The 50% paw withdrawal threshold (grams) was calculated using the ‘up-down’ method (starting with the 0.7 g filament). Three baseline measurements were taken prior to experimentation, the mean of which is presented. Data presented are means of the left and right hind paw thresholds (which show no difference from each other). All behavioural tests were conducted by an experimenter blinded to the treatment regime.

### 2.3 Drug administration

Vincristine sulphate (933 g/mol, Sigma-Aldrich) was dissolved in sterile saline 0.5 mg/kg/day and injected intraperitoneally (i.p.) using a 25-g needle either as a one-off injection (for 24 h experiments) or one cycle (days 0–4 inclusive). RO5461111 (Rupanagudi et al., 2015) and MIV-247 ((S)-N-(1-(2-amino-2-oxoacetyl)cyclobutyl)-2-(2,2-difluoropropanamido)-3-(1-fluorocyclopentyl) propenamide) (Hewitt et al., 2016) were a gift from Medivir and were both administered orally at 200 µmol/kg twice a day for each day of VCR treatment. MIV-247 was supplied pre-formulated in 20% 2-Hydroxypropyl-beta-cyclodextrin (HP-β-CD) that was used as vehicle control and administered at 5 ml/kg (Hewitt et al., 2016). RO54461111 was dissolved in 0.5% methylcellulose in distilled water (that was used as vehicle control) and also administered at 5 ml/kg.

### 2.4 Immunohistochemistry

Mice, under pentobarbital anaesthesia were transcardially perfused with saline and 4% paraformaldehyde (PFA) as described previously (Montague et al., 2018). The lumbar enlargement was excised and post-fixed for 4 h before being dehydrated in 30% sucrose and subsequently embedded in optimum cutting temperature-embedding medium (VWR) and snap frozen on dry ice. Fifteen μm sections were obtained and mounted on Superfrost Plus sides (VWR). Sections were permeabilised for 15 min at room temperature in 0.1% PBS-Triton X-100 (Sigma-Aldrich) then blocked in 0.1% PBS-Triton X-100 + 3% BSA (Sigma-Aldrich) for 1 h at room temperature before being incubated in primary antibody at 4 °C for 16 h. Slides were washed three times in 0.1% PBS-Triton X-100, before being incubated with the appropriate fluorescently tagged secondary antibody for 1.5 h at room temperature. Slides were washed three times again before being incubated for 5 min in DAPI diluted in PBS at 1:2000 before being washed again and then mounted using FluorSave™ (Merck, UK). Slides were visualised under a Zeiss LSM710 confocal microscope (Zeiss). Five sections per mouse were randomly selected and analysed blind as described previously (Old et al., 2014).

### 2.5 Antibodies

For monocyte/macrophages expression anti-Ly6C (1:200 Abcam) was used and followed by anti-rat Alexa 488 (1:1000, Invitrogen). To visualise microglia/macrophages, resident microglia, claudin-5 and ZO-1, anti-Iba1 (1:1000 WAKO), anti-TMEM119 (1:500, Abcam), anti-claudin-5 (1:500, Abcam) and anti-zo-1 (1:500, ThermoFisher scientific) were used, respectively. All were followed by anti-rabbit Alexa 488 (1:1000, Invitrogen). To visualise CatS, anti-CatS (1:100, Santa Cruz) was used followed by anti-mouse Alexa 488 (1:1000, Invitrogen). For FACS analysis reported below the following antibodies were used: occludin monoclonal antibody OC-3F10 (1:100, Invitrogen) P-glycoprotein monoclonal antibody C213 (1:10, Thermo Fisher Scientific). ICAM-1/VCAM-1, anti-mouse CD54 (1:200, e-BioLegend) and anti-mouse CD106 (1:200, e-BioLegend). Secondary coupled antibody used for occludin and p-Glycoprotein was an anti-mouse Alexa 488 (1:200, Life Technology).

### 2.6 Western blot analysis

Sciatic nerves and lumbar spinal cords (dorsal) were fresh-dissected, snap-frozen and stored at −80 °C until use. Tissue was homogenised in ice-cold RIPA buffer (20 mM tris(hydroxymethyl)aminomethane; 10 mM NaF; 150 mM NaCl; 1% nonyl-phenoxylpolyethoxylethanol; 1 mM phenylmethanesulfonyl fluoride; 1 mM Na_3_VO_4_ (all Sigma-Aldrich); and 10 mg/ml proteinase inhibitor (Roche)) before being incubated at 4 °C with agitation for 2 h. Samples were then centrifuged at 4 °C and 13,000 rpm for 20 min and the supernatant obtained was used for Western blot. Protein concentration was determined using the bicinchoninic acid (BCA) protein assay (Pierce). Twenty micrograms of protein per sample was separated on a 15% SDS-PAGE gel (or 8% gel for ZO-1) and transferred onto a nitrocellulose membrane in accordance with manufacturer’s instructions (Bio-Rad, UK). Blots were blocked for 1 h at room temperature in 0.1% TBS-Tween-20 (TBST) + 5% skimmed milk before being incubated in the required primary antibody (CatS at 1:500, Santa Cruz; Claudin-5 at 1:1000, Abcam; ZO-1 at 1:1000, ThermoFisher Scientific) at 4 °C overnight along with a loading control (β-actin (mouse or rabbit) 1:1000, Abcam). After washing in 0.1% TBST five times, blots were incubated for 1 h at room temperature with the appropriate HRP-conjugated secondary antibody (1:2000, DAKO). Blots were washed before being developed with Luminata Forte Western HRP Substrate (Millipore). Bands were visualised using BioSpectrum© Imaging and quantified using Quantity One (Bio-Rad, UK).

### 2.7 *In vivo* imaging

CCR_2_-RFP mice were anaesthetised with an initial dose of urethane (12.5% w/v, 0.3 ml IP). Further doses were administered approximately every 15 min to achieve surgical anaesthetic depth. Throughout the surgery and imaging period mice were maintained close to 37 °C, using a homeothermic heating mat and rectal probe. For increased stability and ease of breathing a tracheal catheter was installed but mice remained freely breathing. An incision was made in the skin above the lumbar enlargement and the spinal column was stabilised on a custom-made stage using spinal camps (Precision Systems and Instrumentation). Vertebrae over L3 and L4 spinal segment were removed in a laminectomy and the exposed spinal cord was cleaned and moistened with saline and covered with silicone elastomer (World Precision Instruments, Ltd). To visualise blood vessels, mice received an intravenous injection of 50 µl dextran fluorescein (12.5 mg/ml; molecular weight: 70,000), prior to imaging. Mice were placed under an Eclipse Ni-E FN upright confocal/multiphoton microscope (Nikon) where the ambient temperature was maintained at 32 °C and core body temperature continued near 37 °C. Images were acquired using a 20× extra-long working distance dry objective. Dextran signal was obtained using a 488 nm Argon ion laser whilst RFP fluorescence was obtained using 561 nm diode laser. To detect the presence of blood vessels a single image of the Dextran and RFP signal was taken before and after time-lapse recording while RFP+ monocyte movement and adhesion was recorded for between 20 and 60 min using only RFP acquisition.

### 2.8 bEND.3 cell culture

The bEND3 (Immortalized Murine Brain Microvascular Endothelial Cell) cell line was obtained from ECACC. They originate from mouse SV129 brain endothelioma.

The bEND3 cells were maintained in Dulbecco’s Modified Eagle Medium (DMEM) 4.5 g/L d-Glucose + GlutaMAX medium (Gibco, Thermo Fisher Scientific, UK) with 10% of foetal bovine serum (FBS-Sigma), 1:100 Non-essential Amino Acid Solution (NEAA) and 1:1000 Gentamicin (Gibco_Thermo Fisher Scientific-UK)

### 2.9 FACS analysis

bEND3 cells (1 × 10^6^) were fixed with 100 μl of 2% paraformaldehyde (PFA) (Sigma-Aldrich, UK) for 10 min at RT. The cells were incubated in 100 µl of primary antibody against the antigens: occludin, P-glycoprotein, ICAM and VCAM in FACS buffer (1% Bovine Serum Albumin in Phosphate buffered saline (PBS) with Ca^2+^ and Mg^2+^) in the dark for 30 min.

When the first antibody was not coupled, cells were further incubated with a secondary antibody (100 µl) for 30 min at RT. Sample were washed in FACS buffer, suspended in 200 µl PBS, and analyzed at the FACS machine (BDFortessa, BD, UK) using a blue laser for occludin and P-glycoprotein Alexa fluor 488 (B530/30), violet for Pacific blues VCAM (V450/50) or Red laser for APC labelled antibody ICAM (R670/14).

### 2.10 Paracellular permeability

bEND3 cells were grown on Transwell polycarbonate filters (pore size, 0.4 μm; Sigma-Aldrich) coated with calf skin collagen type I (Sigma-Aldrich) and paracellular permeability of 70-kDa FITC-dextran was assessed after stimulation as previously described (Dehouck et al., 1992); The permeability coefficient and clearance were calculated from the initial concentration of tracer in the luminal chamber and final concentration in the abluminal chamber: Clearance (FITC) = [C]l * Vl/[C]u where [C]u is the initial luminal tracer concentration, [C]l is the abluminal tracer concentration and Vl is the volume of the abluminal chamber. Concentration values of the lower chamber were calculated by plotting a standard curve of known amounts of FITC-dextran. An increment of cleared volumes was plotted against time of measurement (60 min). The total resistance coefficient opposed to the passage of the dye is the sum of the resistance offered by the monolayer and the one offered by the filter itself (PStot). The slope of the clearance curve with a control membrane was denoted by PSmembrane. The real PS value for the bEND3 monolayer (PStrans) was calculated from 1/PStot. The PStrans values were divided by the surface area of the Transwell inserts to generate the permeability coefficient (Ptrans, in cm/min). Each experimental repeat was performed in duplicate and over at least 3–5 independent experiments.

Transendothelial resistance (TEER) across the monolayer was determined by using an Endohmeter (World Precision Instruments). Resistance from coated cell-free inserts was always subtracted from the resistance obtained in the presence of endothelial cells.

### 2.11 Evans blue injections and extraction from tissue

Evans Blue powder was purchased from Sigma, UK and dissolved in sterile 0.9% saline at a concentration of 50 mg/ml. The solution was then filtered and administered intravenously into the lateral tail vein of C57Bl6 mice at 50 mg/kg. Mice were placed back in their cages and observed regularly before tissue was fresh-dissected at 24 h after injection. Tissue was cut in to small pieces and weighed (approx. 300 mg) and incubated in 500 μl saline for 2 h. Solutions were then centrifuged at 10,000 rpm for 10 min. The supernatants were then treated with 1:2 trichloroacetic acid (TCA) before being centrifuged at 10,000 rpm for 20 min (Wang and Lai, 2014). The final supernatant was then used to measure absorbance at 620 nm and concentration determined using standardized EB solutions (0.025–1000 μg/ml). EB was then expressed as ng/mg of tissue.

## 3 Results

### 3.1 Vincristine upregulates adhesion molecules in CNS endothelial cells *in vitro*

**Fig. 1:**
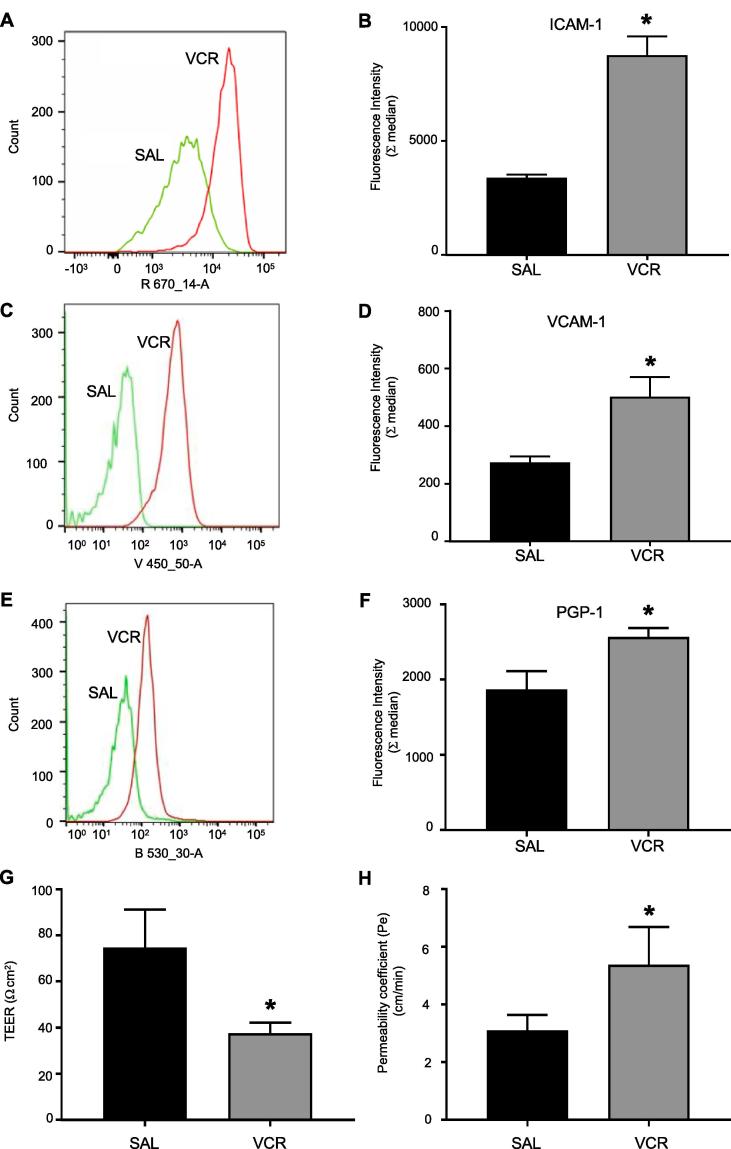
VCR induces activation of CNS endothelial cells. Bend3 cells were incubated with Vincristine (100 nM) or saline control for 24 h. (A) Representative FACS profile showing the intensity of fluorescence for ICAM expression. (B) Quantification of ICAM expression (n = 3 independent experiments). (C) Representative FACS profile showing the intensity of fluorescence for VCAM expression. (D) Quantification of VCAM (n = 3 independent experiments). (E) Representative FACS profile showing the intensity of fluorescence for P-glycoprotein expression. (F) Quantification of PGP-1 expression (n = 3 independent experiments). (G) Trans-endothelial resistance (TEER) (n = 3 independent experiments). (H) Paracellular permeability expressed as permeability coefficient of bEND3 cells treated with VCR. Values are mean ± SEM. Statistical analysis was performed by using Student’s *t* test *p < 0.05 compared to saline treated cells.

In order to elucidate whether VCR could alter permeability of the BSCB, we began with an *in vitro* model of endothelial cells in the murine CNS, and quantified adhesion molecule expression as a read-out of activation. We treated bEND.3 cells with VCR (100 nM) for 24 h and using flow cytometry we observed that both ICAM-1 and VCAM-1 expression was significantly higher (p < 0.05) than under control conditions (Fig. 1A–D). We also observed that the expression of P-glycoprotein in VCR treated cells was significantly higher (p < 0.05) than in unstimulated cells (Fig. 1E–F). Furthermore, VCR treatment significantly decreased (p < 0.05) *trans*-endothelial resistance and increased (p < 0.05) paracellular permeability (Fig. 1G, H) suggesting that VCR treatment alters endothelial functionality.

These data indicate that VCR induces CNS endothelial cell activation, as we previously observed in the context of the periphery using primary HUVECs (Old et al., 2014). In addition, the observation that VCR reduces endothelial resistance and increases permeability of the endothelium *in vitro*, suggests that VCR treatment *in vivo* could result in alteration of vascular permeability and blood cell trafficking in CNS tissue.

### 3.2 Evidence of monocyte trafficking in the spinal cord after VCR systemic treatment

In order to assess whether VCR-induced changes in the endothelium, and thus potential changes in vascular permeability, were associated with blood cell trafficking through the endothelium, we observed monocyte trafficking in mice in which the chemokine receptor CCR_2_ is RFP-tagged and thus CCR_2_^+^ monocytes are labelled with RFP. CCR_2_ is not expressed by microglia (Mizutani et al., 2012), and therefore the presence of mobile, CCR_2_ positive cells can be taken as evidence for monocyte trafficking from the blood stream to the adjacent tissue.

**Fig. 2:**
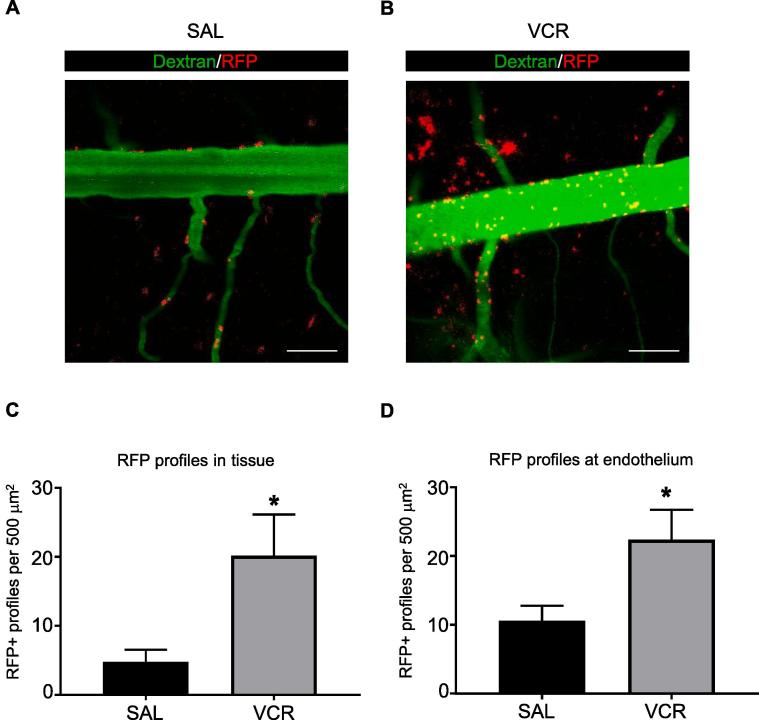
Increased monocyte adhesion to and infiltration into the spinal cord 24 h after one dose of VCR. (A-B) Representative images from a time series of spinal cords taken from saline (A) and VCR-treated (B) mice. Blood vessels are labelled with dextran fluorescein (green) and monocytes are tagged with CCR_2_-RFP (red). Scale bar = 100 μm. (C) Quantification of the number of CCR_2_-RFP+ profiles observed outside of the vessel. One dose of VCR significantly increases RFP+ profiles found in spinal cord tissue (mean ± SEM, n = 5 mice per group). *p < 0.05, Student’s paired *t*-test. (D) Quantification of the number of CCR_2_-RFP+ profiles observed at the endothelium. One dose of VCR significantly increases RFP+ profiles found in spinal cord tissue (mean ± SEM, n = 5 mice per group). *p < 0.05, Student’s paired *t*-test. (For interpretation of the references to colour in this figure legend, the reader is referred to the web version of this article.)

CCR_2_-RFP mice were injected with VCR and *in vivo* imaging was performed in the spinal cord 24 h later. We have previously observed that mechanical hypersensitivity occurs within 24 h of the first dose of VCR and that *in vitro*, the endothelium is activated 24 h after VCR treatment *in vitro* and so we attempted to investigate if monocyte infiltration into the spinal cord as a result of alterations in the endothelium could play a role in hypersensitivity at this timepoint. In order to visualise blood vessels and hence distinguish between monocytes that were present in blood and those that were present in tissue, we injected dextran fluorescein intravenously prior to imaging. We found that 24 h after a single administration of VCR, a significantly higher number of RFP+ cells could be observed outside of the blood vessels i.e. in spinal cord tissue. Specifically, in VCR-treated mice we found approximately four times more RFP+ cells in spinal cord tissue compared to controls (p < 0.05) (Fig. 2A–C). Furthermore, we also observed preliminary evidence for significantly more (p < 0.05) adhesion of RFP+ monocytes at the vessel wall in VCR-treated mice (Fig. 2D). Indeed, in VCR-treated mice we observed a 2-fold increase in the number of monocytes located at the endothelium relative to controls suggesting a link between up-regulation of endothelial adhesion molecules that we observed *in vitro*, and significant increase in apparent cell adhesion.

These data indicate that monocytes are likely to infiltrate the spinal cord after a single dose of VCR, suggesting that endothelial activation at the BSCB could have occurred, corroborating the *in vitro* observations obtained in endothelial cells.

### 3.3 Alterations in vascular permeability at the end of one cycle of VCR systemic treatment

**Fig. 3:**
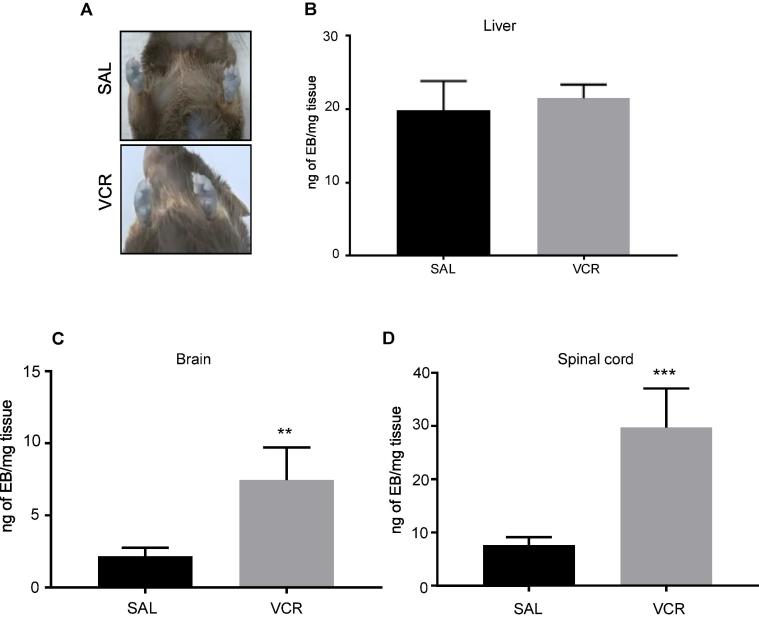
One cycle of VCR treatment significantly increases vascular permeability in brain and spinal cord. (A) Observation of paws prior to tissue harvesting showed no obvious difference in Evans Blue (EB) accumulation between saline-treated and VCR-treated mice. (B) Quantification of EB (expressed as ng/mg of tissue) in liver tissue from saline- and VCR-treated mice (mean ± SEM, n = 7–8 mice per group). (C,D) Quantification of EB in brain (C) and spinal cord (D) from saline- and VCR-treated mice (mean ± SEM, n = 7–8 mice per group). **p < 0.01, ***p < 0.005, one-way ANOVA, post-hoc Tukey test. (For interpretation of the references to colour in this figure legend, the reader is referred to the web version of this article.)

To determine whether changes in vascular permeability in the CNS occur *in vivo*, and following a more clinically-relevant dosing regime, we treated mice with one cycle of VCR (5 days) and subsequently assessed Evans Blue (EB) extravasation. Comparable EB amounts were observed in paws prior to tissue harvesting (Fig. 3A) and likewise similar levels were found in liver tissue from VCR-treated and control mice (Fig. 3B) indicating that EB delivery and extraction were. Following VCR treatment, we did however measure a significant increase in the amount of detectable EB in both brain and spinal cord tissue, relative to controls (Fig. 3C-D). Specifically, in brain tissue from VCR-treated mice, the amount of EB that could be measured per mg of tissue was more than three times higher (p < 0.01) than that measured in brain tissue from control mice that had been treated with saline for 5 days (Fig. 3C). Likewise, when we measured EB in spinal cord tissue, levels were almost four times greater (p < 0.005) in VCR-treated mice than in tissue from controls (Fig. 3D). These data suggest that CNS vascular permeability is selectively increased during VCR treatment.

### 3.4 Disruption of tight junction protein expression and presence of infiltrating monocytes in the spinal cord after VCR treatment

**Fig. 4:**
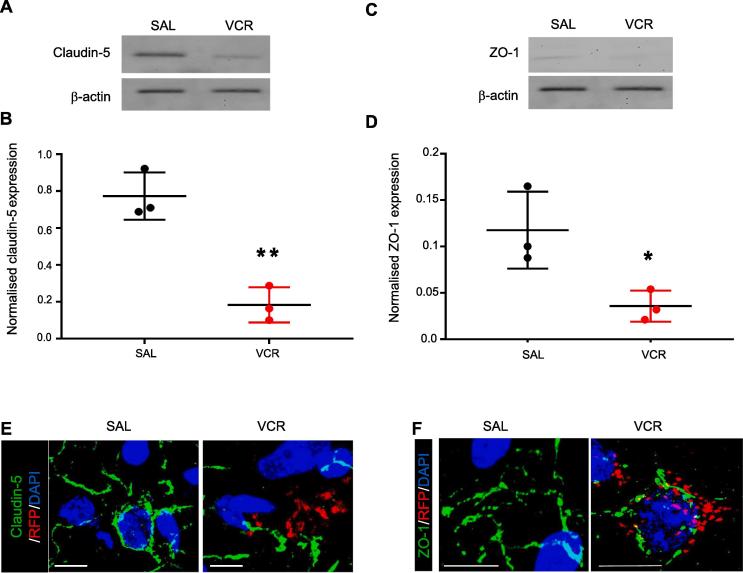
One cycle of VCR treatment disrupts expression of tight junction proteins in the spinal cord. (A and C) Representative blots of either Claudin-5 (A, 24 kDa) or ZO-1 (B, 187 kDa) and β -actin loading control (42 kDa) in lumbar spinal cord dorsal horn homogenates obtained from wild-type mice treated with one cycle of VCR or saline controls (days 0–4 inclusive). (B and D) Quantification of claudin-5 (B) and ZO-1 (D) band density normalised to loading control. VCR treatment significantly decreases the expression of both proteins in spinal cord homogenates. (n = 3 mice). *p < 0.05, **p < 0.01, one-way ANOVA, post-hoc Tukey test. (E) Representative images of claudin 5 (green) and CCR_2_ (RFP, red) in lumbar spinal cord sections (dorsal horn) from saline-treated and VCR-treated mice. Nuclear stain DAPI in blue. (F) Representative images of ZO-1 (green) and CCR_2_ (RFP, red) in lumbar spinal cord sections (dorsal horn) from saline-treated and VCR-treated mice. Nuclear stain DAPI in blue Scale bars in panels in E and F = 10 μm. (For interpretation of the references to colour in this figure legend, the reader is referred to the web version of this article.)

We then further assessed the integrity of the BSCB after a cycle of VCR treatment by evaluating tight junction protein expression in order to assess whether their disruption could account for the increased EB permeability observed at this time point. We used Western blot analysis and immunohistochemistry in order to assess the expression of claudin-5 and ZO-1, and their assembly on the endothelium in the murine spinal cord. We observed that at the end of one cycle of VCR treatment, both claudin-5 and ZO-1 expression were significantly decreased (p < 0.01 and 0.05, respectively) relative to the expression levels observed in spinal cord tissue from saline-treated control mice (Fig. 4A–D). In addition, immunohistochemistry indicated that claudin-5 and ZO-1 organization was fragmented and that CCR_2_/RFP+ profiles were clustered in regions where tight junction integrity was suggested to be disrupted (Fig. 4E–F).

We then investigated whether altered vascular permeability and potential changes in tight junctions resulted in infiltration of monocytes into the spinal cord following one cycle of VCR treatment, which was suggested to occur from as soon as 24 h after the first treatment as demonstrated by our intravital data.

**Fig. 5:**
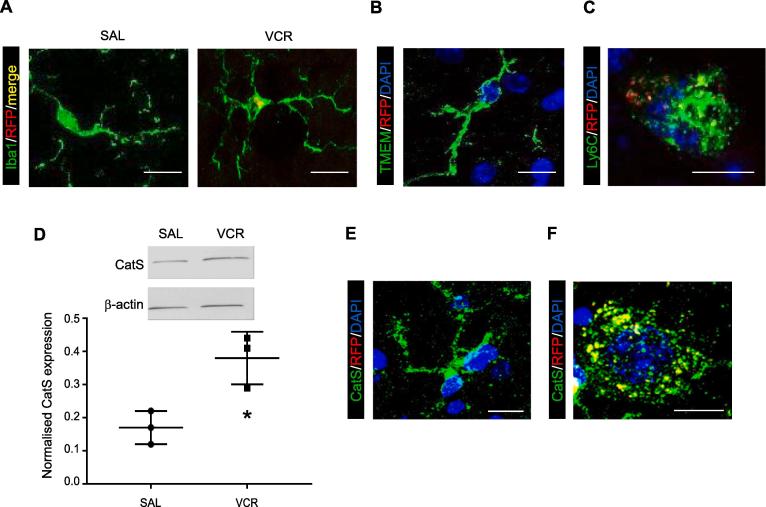
One cycle of VCR treatment results in monocytes/macrophages infiltration and CatS elevation in the spinal cord. (A) Representative images showing microglia/macrophage marker Iba1 (green) in saline-treated and VCR-treated lumbar spinal cord dorsal horn from CCR_2_-RFP heterozygous mice. CCR_2_ immunoreactivity (RFP, red, merge yellow) is only found in Iba1+ cells in spinal cords from VCR-treated mice. (B) Representative image showing TMEM119 immunoreactivity (green, resident microglia marker). No CCR_2_ (RFP, red) immunoreactivity is found in TMEM119+ cells. Nuclear marker DAPI in blue. (C) Representative image showing Ly6C immunoreactivity (green, monocyte/macrophage marker). CCR_2_ (RFP, red) immunoreactivity is found in Ly6C+ cells. Nuclear marker DAPI in blue. (D) Representative blot and quantification of CatS (37 kDa) and β -actin loading control (42 kDa) in lumbar spinal cord dorsal horn homogenates obtained from wild-type mice treated with one cycle of VCR (or saline controls). One cycle of VCR significantly increases CatS expression. (n = 3 mice). *p < 0.05, one-way ANOVA, Tukey test. (E,F) Representative image showing CatS immunoreactivity (green) in lumbar spinal cord dorsal horn from mice treated with one cycle of VCR. CCR_2_ (RFP, red) immunoreactivity is only found in CatS+ cells (yellow) with a phagocytic phenotype. Nuclear marker DAPI in blue. Scale bar in all panels = 10 μm. (For interpretation of the references to colour in this figure legend, the reader is referred to the web version of this article.)

We detected the presence of RFP(CCR_2_)^+^/Iba1^+^ cells in the lumbar dorsal horn from VCR-treated mice (Fig. 5A). In the spinal cord Iba1 immunoreactivity is present on both resident microglia and infiltrated monocytes/macrophages. However, microglia do not express CCR_2_ (Mizutani et al., 2012) and thus it is possible that RFP+/Iba1+ cells that are observed in the spinal cord are infiltrated monocytes/ macrophages. Indeed, we could not detect RFP signal in cells that were positive for TMEM119, a specific marker for resident microglia (Fig. 5B). RFP signal however was detectable within TMEM119-stained sections, thus the lack of RFP signal in TMEM119 cells was not due to a complete lack of detectable RFP signal (Suppl. Fig. 1). Furthermore, we were also able to observe the presence of RFP+ cells that were also positive for the monocyte/macrophage precursor marker Ly6C in the dorsal horn of lumbar spinal cord from VCR-treated mice but not from saline-treated mice after one VCR treatment cycle (Fig. 5C and Suppl. Fig. 2B). Our immunohistochemical data therefore indicate that monocytes infiltrate into the spinal cord in the first VCR treatment cycle.

### 3.5 Functional role for infiltrating monocytes following VCR systemic treatment

As we are interested in a functional role for infiltrating monocytes in the development of VCR-induced nociception, we postulated that these cells would provide a source of the pro-nociceptive enzyme CatS in the spinal cord. Indeed, monocytes/macrophages-derived CatS exerts a pro-nociceptive action in the periphery in models of neuropathic pain (Barclay et al., 2007, Zhao et al., 2014).

Following one cycle of VCR treatment we observed that the expression of CatS in the lumbar spinal cord significantly increased (p < 0.05), as demonstrated by Western blot analysis (Fig. 5D). Furthermore, not only did we observe CatS immunoreactivity in Iba1+ cells, which is expected since CatS is normally expressed by microglia (Fig. 5E), we also detected CatS immunoreactivity in RFP+ cells that possessed a morphology associated with phagocytic cells such as macrophages (Fig. 5F).

Taken together, these data suggest that monocytes expressing CatS infiltrate into the spinal cord following VCR treatment and provide an additional source of the enzyme alongside the microglia. It is plausible that monocytes/macrophages release CatS instead of microglia, which are not activated following VCR treatment (Old et al., 2014). In order to validate the biological significance of infiltrating monocyte-derived CatS, we investigated whether administration of a CatS inhibitor affected the development of allodynia.

### 3.6 A centrally-penetrant Cathepsin s inhibitor is effective in reducing VCR-induced nociception

In light of the data presented so far, we postulated that monocytes infiltrate into the spinal cord during VCR treatment and once there, could contribute to allodynia via the release of CatS that liberates neuronal fractalkine, thus activating CX_3_CR_1_ receptors that are constitutively expressed by microglia (Clark et al., 2007).

**Fig. 6:**
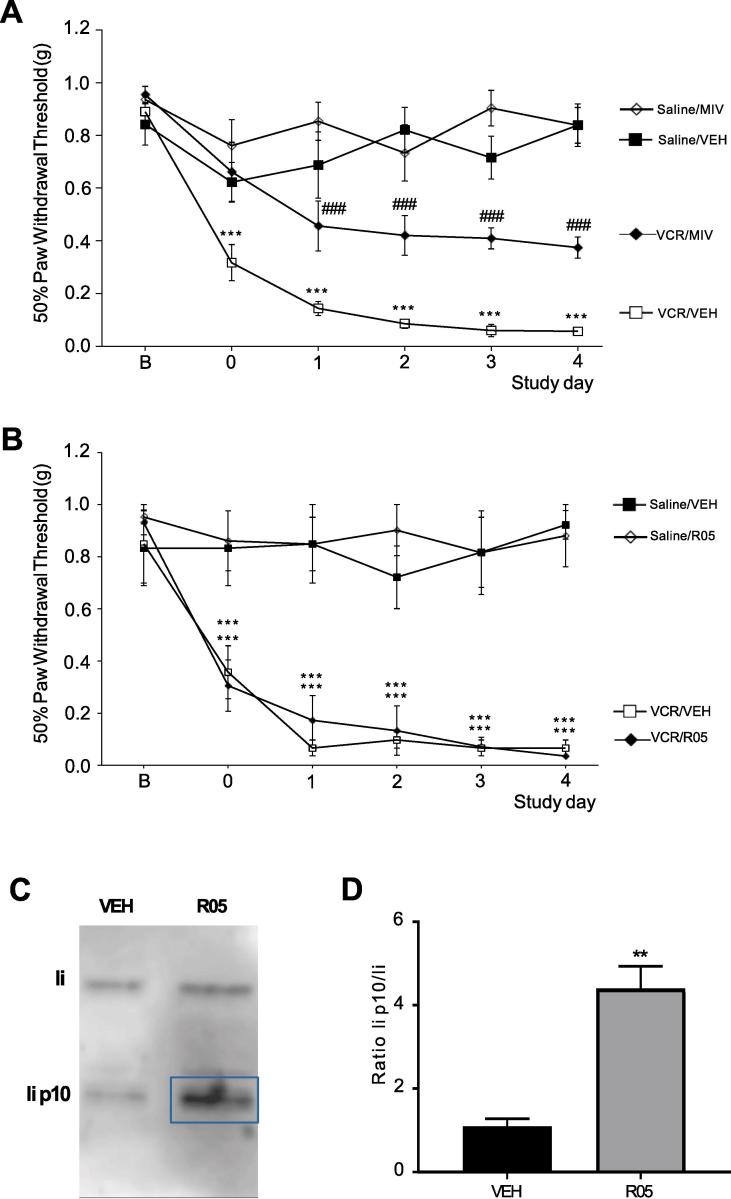
CatS inhibitors reduce VCR-induced mechanical hypersensitivity only when centrally-penetrant. (A) Mechanical thresholds are significantly higher in VCR-treated male and female mice (days 0–4 inclusive), which also received the centrally-penetrant MIV-247 (MIV) (200 µmol/kg p.o. twice a day, every day) alongside VCR than in VCR/vehicle-treated mice. Data expressed as 50% paw withdrawal thresholds (mean ± SEM, n = 7 mice per group, 4 female and 3 male) measured 3 h after administration. ***p < 0.001 compared to Saline/VEH group; ###p < 0.001 compared to VCR/VEH group, Repeated Measures ANOVA, post hoc Tukey test. (B) Mechanical thresholds in mice treated with VCR (days 0–4 inclusive) are not altered 3 h after administration of the peripherally-restricted RO5461111 (RO5) (200 µmol/kg p.o. twice a day, every day) alongside VCR. Data expressed as 50% paw withdrawal thresholds (mean ± SEM, n = 7 mice per group). ***p < 0.001 compared to Saline/VEH and Saline/RO5 groups Repeated Measures ANOVA, post hoc Tukey test. (C-D) Representative blot (C) and quantification (D) of li p10 fragment expression relative to li in snap frozen spleen from R05-treated and control mice (3 h after administration) (n = 3 mice ± SEM) **p < 0.01, Student’s *t*-test.

The administration of a centrally-penetrant CatS inhibitor (MIV-247) (Hewitt et al., 2016) significantly (p < 0.001) reduced VCR-induced mechanical hypersensitivity within 24 h of the first dose being administered and this effect persisted throughout the first VCR treatment cycle (Fig. 6A). However, equimolar administration of a peripherally-restricted CatS inhibitor (RO5461111) to VCR-treated mice during the first treatment cycle did not delay the onset or reduce the severity of VCR-induced mechanical hypersensitivity. Indeed, there was no difference in the paw withdrawal thresholds between VCR-treated mice who had received RO5461111 and those that had received the vehicle (Fig. 6B). To ensure that this lack of effect of RO5461111 was due to the antagonist's peripheral restriction and not to an insufficient dose or poor oral bioavailability, we tested CatS target engagement for the antagonist in spleen tissue as a positive control. In the presence of a CatS inhibitor, the cleavage of Lip P10 (P10) in B-lymphocytes and dendritic cells is blocked, resulting in accumulation of P10. Indeed, in spleen homogenates isolated from RO5461111-treated mice, we observe significant P10 accumulation (p < 0.01) suggesting that the antagonist is biologically active in the periphery under these experimental conditions (Fig. 6C, D).

The observation that VCR-induced nociception was only significantly reduced when a centrally-penetrant CatS antagonist was used, does suggest that CatS plays a role centrally in mediating VCR mechanisms.

## 4 Discussion

**Fig. 7:**
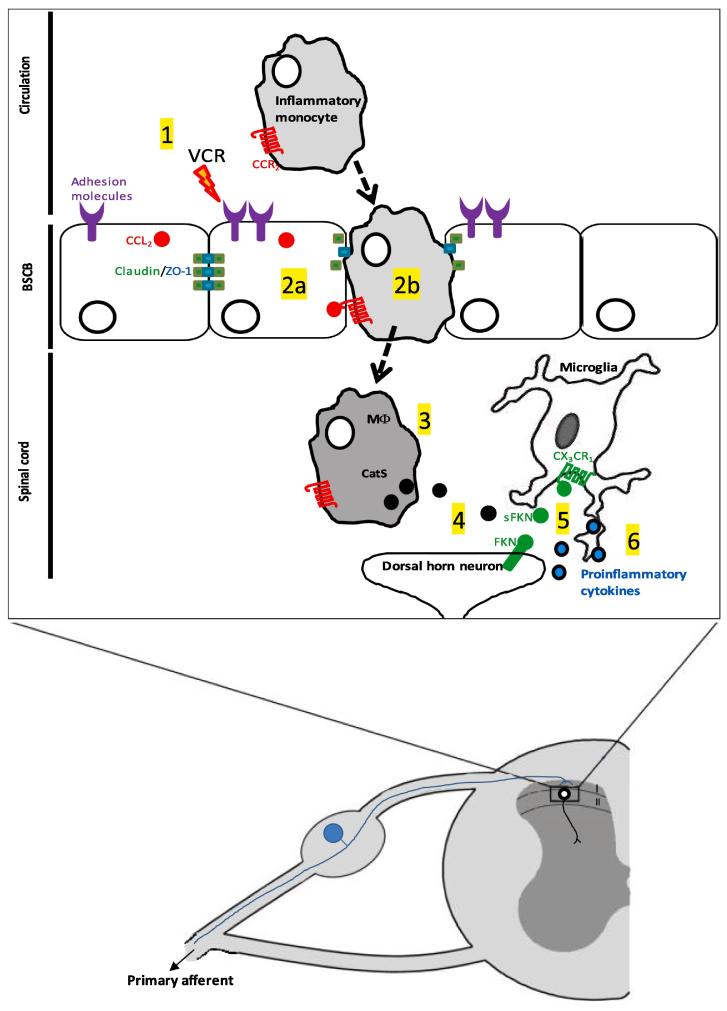
Changes in vascular permeability following VCR treatment result in the infiltration of monocytes, which play a role in central mechanisms of pain. 1) Following treatment with vincristine (VCR), endothelial cells at the blood-spinal cord barrier upregulate adhesion molecules and are thus activated. 2a) VCR treatment also disrupts tight junction organization between endothelial cells and so monocytes, (which express CCR_2_ and are thus attracted by endothelial-expressed CCL_2)_, are able to infiltrate into the spinal cord (2b). 3) Infiltrating monocytes provide a source of cathepsin S (CatS). 4) Cat S cleaves fractalkine (FKN) expressed by dorsal horn neurons. 5) Soluble FKN (sFKN) activates CX_3_CR_1_ receptors expressed by microglia, which in turn release proinflammatory, pronociceptive cytokines (6).

Chemotherapy-induced neuropathic pain is a dose-limiting side effect of many cancer therapies that has the capacity to diminish their success (Seretny et al., 2014). It is therefore critical that our understanding of the underlying mechanisms of chemotherapy pain is advanced and thus the development of new, more effective therapies for pain is aided. We elucidated a previously unappreciated pathway by which changes in vascular permeability in the spinal cord following VCR treatment result in infiltration of CCR_2_+ monocytes that contribute to allodynia via the pro-nociceptive enzyme CatS (Fig. 7).

This phenomenon provides a potential means by which communication between peripheral immune cells and dorsal horn neurons/sensory neuron terminals in the spinal cord mediates nociceptive hypersensitivity during VCR treatment.

We uncover a potential role for monocytes expressing CatS in the development of VCR-induced allodynia with CatS activity being more critical in the spinal cord than in the peripheral nerve. Specifically, we show that VCR-induced allodynia is significantly prevented by administration of a centrally penetrant, but not by a peripherally-restricted, CatS inhibitor. We have previously shown that in peripheral nerves VCR-induced activation of the endothelium results in infiltration of monocytes/macrophages. In these cells the activation of CX_3_CR_1_ receptors by FKN results in production of ROS, which can activate TRPA_1_ channels and transmit pain signalling (Old et al., 2014). Although we know that CatS enzymatically cleaves FKN (Clark et al., 2007, Clark et al., 2009), solubilisation of endothelial FKN in peripheral nerves does not appear to contribute to VCR-induced allodynia. Indeed we found that a peripherally-restricted CatS antagonist is devoid of anti-nociceptive effects. We discounted the possibility that this was due to lack of compound bioavailability and *in vivo* activity by confirming P10 accumulation in spleen. In contrast, the significant anti-nociceptive effect of a centrally-penetrant CatS inhibitor indicates that CatS derived from a central source plays a role in VCR-induced allodynia.

In the CNS, CatS is expressed and released by microglia (Clark et al., 2007, Clark et al., 2010). However, changes in microglial number and morphology are not apparent in preclinical chemotherapy models (Old et al., 2014) suggesting that the microglial response is relatively mild and they are therefore unlikely to release significant amounts of CatS during VCR treatment. We suggest that monocytes/macrophages in the spinal cord provide a source of CatS, which we know can solubilise neuronal FKN that activates CX_3_CR_1_ receptor constitutively expressed by microglia.

Specifically, we delineated a mechanistic pathway that starts with a systemic VCR-mediated increase in vascular permeability and endothelial activation in the spinal cord, which are followed by the consequential infiltration of monocytes/macrophages. We provide evidence for VCR’s ability to induce up-regulation of endothelial adhesion molecules, increase CNS vascular permeability and reduce cell resistance. We corroborate these *in vitro* data with an *in vivo* series of data that demonstrate increase of vascular permeability, monocyte trafficking and an increased in CatS expression in the spinal cord after VCR treatment.

Indeed, we found evidence that permeability of the spinal cord and brain is elevated at the end of VCR treatment as indicated by EB extravasation. Within 24 h of VCR treatment we found upregulation of adhesion molecules in endothelial cells *in vitro*, whilst *in vivo* we observed a significant increase in monocytes (CCR_2_^+^ cells) in spinal cord tissue as well as a significant increase in cells adhering to the vessel wall, which is the first step of monocyte infiltration (Gerhardt and Ley, 2015). Due to practical limitations however, the time series that we obtained were not long enough to observe infiltration, which would take hours longer than the maximum of 1 h that we were able to obtain.

Furthermore, we found evidence to suggest that tight junction integrity is disrupted in the spinal cord of VCR-treated mice. Specifically, we observed a decrease in the expression of the tight junction proteins claudin-5 and ZO-1 in lumbar dorsal spinal cord as well as a change in protein organization as suggested by our immunohistochemical data. Therefore, alterations in permeability of the BSCB at the end of one VCR treatment cycle could be a result of changes in tight junction protein expression. Furthermore, our observation the CCR_2_/RFP^+^ profiles accumulated in regions where tight junction protein organization appeared to be disrupted suggests that infiltration of monocytes/macrophages could be aided by the disruption of endothelial tight junctions in the spinal cord.

Indeed, we observed the presence of Iba1-positive cells that were also positive for CCR_2_/RFP in the lumbar spinal cord. Both microglia and monocytes/macrophages express Iba1 and yet microglia do not express CCR_2_ (Mizutani et al., 2012) and we observed no RFP signal in cells that were positive for the microglial marker TMEM119. This suggests that the origin of Iba1^+^/RFP^+^ cells in the spinal cord following VCR treatment is not microglia and indeed we observe cells that are positive for the monocyte/macrophage precursor marker; Ly6C in the spinal cord following one cycle of VCR treatment that are also positive for CCR_2_/RFP. It is also very likely that infiltrating monocytes/macrophages provide a source of CatS, as we also observe an increase in CatS protein expression after one cycle of VCR.

Finally, it is worth noting that endothelial cells in the CNS do not express FKN (Kim et al., 2011), therefore soluble FKN is unlikely to be the mechanism that accounts for CX_3_CR_1_-expressing monocyte trafficking in this case.

Overall our study highlights the significance of the interaction between peripheral immune cells and nociceptive neurons in the spinal cord, which mediates nociceptive hypersensitivity during VCR treatment, a potential prototype for other chemotherapy drugs. Obtaining a better understanding of alterations in vascular permeability that occur as a result of VCR treatment begins to guide us towards a better understanding of potential central mechanisms that regulate pain associated this pain.

## References

[b0005] Abbott N.J., Patabendige A.A., Dolman D.E., Yusof S.R., Begley D.J. (2010). Structure and function of the blood-brain barrier. Neurobiol. Dis..

[b0010] Balayssac D., Cayre A., Authier N., Bourdu S., Penault-Llorca F., Gillet J.P., Maublant J., Eschalier A., Coudore F. (2005). Patterns of P-glycoprotein activity in the nervous system during vincristine-induced neuropathy in rats. J. Peripher. Nerv. Syst..

[b0015] Barclay J., Clark A.K., Ganju P., Gentry C., Patel S., Wotherspoon G., Buxton F., Song C., Ullah J., Winter J., Fox A., Bevan S., Malcangio M. (2007). Role of the cysteine protease cathepsin S in neuropathic hyperalgesia. Pain.

[b0020] Clark A.K., Yip P.K., Grist J., Gentry C., Staniland A.A., Marchand F., Dehvari M., Wotherspoon G., Winter J., Ullah J., Bevan S., Malcangio M. (2007). Inhibition of spinal microglial cathepsin S for the reversal of neuropathic pain. Proc. Natl. Acad. Sci. USA.

[b0025] Clark A.K., Yip P.K., Malcangio M. (2009). The liberation of fractalkine in the dorsal horn requires microglial cathepsin S. J. Neurosci..

[b0030] Clark A.K., Wodarski R., Guida F., Sasso O., Malcangio M. (2010). Cathepsin S release from primary cultured microglia is regulated by the P2X7 receptor. Glia.

[b0035] Clark A.K., Gruber-Schoffnegger D., Drdla-Schutting R., Gerhold K.J., Malcangio M., Sandkühler J. (2015). Selective activation of microglia facilitates synaptic strength. J. Neurosci..

[b0040] Costigan M., Scholz J., Woolf C.J. (2009). Neuropathic pain: a maladaptive response of the nervous system to damage. Annu. Rev. Neurosci..

[b0045] Dehouck B., Dehouck M.P., Fruchart J.C., Cecchelli R. (1992). Upregulation of the low density lipoprotein receptor at the blood-brain barrier: intercommunications between capillary endothelial cells and astrocytes. J. Cell. Biol..

[b0050] Evans T.A., Barkauskas D.S., Myers J.T., Hare E.G., You J.Q., Ransohoff R.M., Huang A.Y., Silver J. (2014). High-resolution intravital imaging reveals that blood-derived macrophages but not resident microglia facilitate secondary axonal dieback in traumatic spinal cord injury. Exp. Neurol..

[b0055] Findley M.K., Koval M. (2009). Regulation and roles for claudin-family tight junction proteins. IUBMB Life.

[b0060] Gerhardt T., Ley K. (2015). Monocyte trafficking across the vessel wall. Cardiovasc. Res..

[b0065] Hewitt E., Pitcher T., Rizoska B., Tunblad K., Henderson I., Sahlberg B.L., Grabowska U., Classon B., Edenius C., Malcangio M., Lindstrom E. (2016). Selective cathepsin S inhibition with MIV-247 attenuates mechanical allodynia and enhances the antiallodynic effects of gabapentin and pregabalin in a mouse model of neuropathic pain. J. Pharmacol. Exp. Ther..

[b0070] Innes S., Pariante C.M., Borsini A. (2019). Microglial-driven changes in synaptic plasticity: a possible role in major depressive disorder. Psychoneuroendocrinology.

[b0075] Kim K.-W., Vallon-Eberhard A., Zigmond E., Farache J., Shezen E., Shakhar G., Ludwig A., Lira S.A., Jung S. (2011). In vivo structure/function and expression analysis of the CX3C chemokine fractalkine. Blood.

[b0080] Mizutani M., Pino P.A., Saederup N., Charo I.F., Ransohoff R.M., Cardona A.E. (2012). The fractalkine receptor but not CCR2 is present on microglia from embryonic development throughout adulthood. J Immunol.

[b0085] Montague K., Malcangio M. (2017). The therapeutic potential of monocyte/macrophage manipulation in the treatment of chemotherapy-induced painful neuropathy. Front. Mol. Neurosci..

[b0090] Montague K., Simeoli R., Valente J., Malcangio M. (2018). A novel interaction between CX3CR1 and CCR2 signalling in monocytes constitutes an underlying mechanism for persistent vincristine-induced pain. J. Neuroinflammation.

[b0095] Old E.A., Nadkarni S., Grist J., Gentry C., Bevan S., Kim K.W., Mogg A.J., Perretti M., Malcangio M. (2014). Monocytes expressing CX3CR1 orchestrate the development of vincristine-induced pain. J. Clin. Invest..

[b0100] Pitcher T., Sousa-Valente J., Malcangio M. (2016). The monoiodoacetate model of osteoarthritis pain in the mouse. J. Vis. Exp..

[b0105] Rupanagudi K.V., Kulkarni O.P., Lichtnekert J., Darisipudi M.N., Mulay S.R., Schott B., Gruner S., Haap W., Hartmann G., Anders H.J. (2015). Cathepsin S inhibition suppresses systemic lupus erythematosus and lupus nephritis because cathepsin S is essential for MHC class II-mediated CD4 T cell and B cell priming. Ann. Rheum. Dis..

[b0110] Santa-Cecília F.V., Ferreira D.W., Guimaraes R.M., Cecilio N.T., Fonseca M.M., Lopes A.H., Davoli-Ferreira M., Kusuda R., Souza G.R., Nachbur U., Alves-Filho J.C., Teixeira M.M., Zamboni D.S., Cunha F.Q., Cunha T.M. (2019). The NOD2 signaling in peripheral macrophages contributes to neuropathic pain development. Pain.

[b0115] Seretny M., Currie G.L., Sena E.S., Ramnarine S., Grant R., MacLeod M.R., Colvin L.A., Fallon M. (2014). Incidence, prevalence, and predictors of chemotherapy-induced peripheral neuropathy: a systematic review and meta-analysis. Pain.

[b0120] Shen S., Lim G., You Z., Ding W., Huang P., Ran C., Doheny J., Caravan P., Tate S., Hu K., Kim H., McCabe M., Huang B., Xie Z., Kwon D., Chen L., Mao J. (2017). Gut microbiota is critical for the induction of chemotherapy-induced pain. Nat. Neurosci..

[b0125] Varvel N.H., Neher J.J., Bosch A., Wang W., Ransohoff R.M., Miller R.J., Dingledine R. (2016). Infiltrating monocytes promote brain inflammation and exacerbate neuronal damage after status epilepticus. Proc. Natl. Acad. Sci. USA.

[bib146] Wang H.-L., Lai T. (2014). Optimization of Evans blue quantitation in limited rat tissue samples. Sci. Rep..

[b0130] Wang F., Zhou F., Kruh G.D., Gallo J.M. (2010). Influence of blood-brain barrier efflux pumps on the distribution of vincristine in brain and brain tumors. Neuro Oncol..

[b0135] Zhang H., Li Y., de Carvalho-Barbosa M., Kavelaars A., Heijnen C.J., Albrecht P.J., Dougherty P.M. (2016). Dorsal root ganglion infiltration by macrophages contributes to paclitaxel chemotherapy-induced peripheral neuropathy. J. Pain.

[b0140] Zhao P., Lieu T., Barlow N., Metcalf M., Veldhuis N.A., Jensen D.D., Kocan M., Sostegni S., Haerteis S., Baraznenok V., Henderson I., Lindström E., Guerrero-Alba R., Valdez-Morales E.E., Liedtke W., McIntyre P., Vanner S.J., Korbmacher C., Bunnett N.W. (2014). Cathepsin S causes inflammatory pain via biased agonism of PAR2 and TRPV4. J. Biol. Chem..

[b0145] Zheng F.Y., Xiao W.-H., Bennett G.J. (2011). The response of spinal microglia to chemotherapy-evoked painful peripheral neuropathies is distinct from that evoked by traumatic nerve injuries. Neuroscience.

